# The Kraft Heinz Company global nutrition targets for the innovation and reformulation of food and beverages: Current and future directions

**DOI:** 10.3389/fnut.2023.1104617

**Published:** 2023-02-02

**Authors:** Lynn Yu, Véronique Braesco, Sheri L. Cooper, Adam Drewnowski, Bridget Hannon Esteves, Andrea Luigi Budelli

**Affiliations:** ^1^The Kraft Heinz Company, Chicago, IL, United States; ^2^VAB Nutrition, Clermont-Ferrand, France; ^3^School of Medical and Health Sciences, Edith Cowan University, Joondalup, WA, Australia; ^4^Center for Public Health Nutrition, University of Washington, Seattle, WA, United States; ^5^The Kraft Heinz Company, Milan, Italy

**Keywords:** reformulation, innovation, renovation, nutrient profiling, The Kraft Heinz Company

## Abstract

Reformulating packaged foods has the potential to improve the nutrient density of the global diet. The present perspective illustrates The Kraft Heinz Company’s approach to product (re)formulation to develop healthier product lines that are lower in saturated fats, total sugars, and sodium, and contain health promoting components. Here we present the rationale for The Kraft Heinz Company’s global nutrition targets used for the global innovation and renovation of foods and beverages. The global nutrition targets use a category specific approach to set maximum levels for the main nutrients of public health concern: saturated fat, total sugars and sodium, taking into account product characteristics (typical portion size, eating occasion, role in the diet, etc.) as well as regulatory, technological, sensory and safety constraints. Benchmarking examples illustrate how the nutrition targets are positioned within the United States, France, and Australia. These global nutrition targets serve as part of The Kraft Heinz Company’s environmental, social and governance nutrition commitments and demonstrates how the food industry is improving the nutritional value of packaged foods and beverages both now and into the future.

## 1. Introduction

Reformulating packaged and processed foods has the potential to improve the nutrient density of the global diet (1). Nutrient density refers to the amount of nutrients a food contains per unit of energy it provides and nutrient dense foods are those high in nutrients but relatively low in energy (2). Increasing rates of overweight and obesity represent a form of malnutrition and are a driver for diet related chronic diseases, such as cardiovascular disease, type 2 diabetes and some types of cancer (3). The World Health Organization (WHO) has encouraged product reformulation by the food industry, with an emphasis on reducing energy and removing excess fat, sugar and salt/sodium from the global food supply (4). A review of global dietary consumption patterns (5), highlights the importance of promoting those dietary components that are currently consumed in suboptimal amounts. The leading dietary risk factors for mortality were diets high in sodium, and low in wholegrains, nuts, seeds, vegetables, fruit and omega-3 fatty acids (5).

Local and regional regulatory bodies, including Public Health England (6), the Food and Drug Administration in the US (7), the French Ministry of Agriculture and Food (8), and the Federal Government of Australia (9) have issued guidance to the food industry regarding reformulation targets for specific food categories. Multiple agencies were behind the elimination of trans fats from the global food supply (10). Reformulation can take the form of reducing added sugars, saturated fat, and sodium in foods or the addition to foods of vitamins, minerals, and other health promoting components (11). Nutrient density can also be improved by the addition of desirable, nutrient-rich food ingredients to product formulations, such as whole grains, nuts, seeds, vegetables, legumes, or fruits, and by the addition of vitamins and minerals. Fortification of foods, and the addition of plant proteins, whole grains, and other whole ingredients to foods are also features of product reformulation.

Food and beverage manufacturers are using a variety of quantitative tools to develop reformulation targets and to benchmark their progress toward healthier product lines (12, 13). Among those are nutrient profiling (NP) methods that capture the overall nutritional value of foods. NP models have provided the scientific basis for many educational, regulatory, and reformulation initiatives. The goal of early NP models was to assess the relative healthfulness of packaged beverages and foods (14), identify those with highest nutritional value and so prompt healthier choices at the point of sale. These NP models were “across-the-board” – meaning that the same nutrient standards were applied to all food groups.

Today NP methods have been further developed by the food industry to provide both direction and benchmarking for product innovation and (re)formulation. Among industry-driven NP models are those developed by Unilever (now Choices) (15), Nestlé (13), PepsiCo (16), Ajinomoto (17) and others. These models are typically category specific, with categories sometimes corresponding to product lines. Category-specific nutrient standards are viewed as better suited to the setting of objectives for product reformulation. The goal is to develop within each food category new or improved products that provide maximum nutritional value and are “best in class.”

Ongoing self-review of nutrient density of the global food supply has been spurred by the Access to Nutrition Initiative (ATNI) (18). ATNI has reviewed product portfolios of major food manufacturers since 2013 and in 2022 conducted its first assessment of all major food retailers within the United Kingdom market. Within the dimension of product (re)formulation, food manufacturers are evaluated based on whether: (1) they have developed a NP model; (2) have used the NP model to screen their products for nutritional value; and (3) have taken steps to assure transparency by publishing and disseminating their nutrient standards and criteria for innovation and renovation.

The present perspective addresses The Kraft Heinz Company’s (KHC) global product portfolio which is composed of 51 separate product categories that align with over 200 brands that are sold in nearly 200 countries. KHC has developed global nutrition targets for each product category, focusing on upper limits for energy, sodium, total sugar, and saturated fat. These standards have been used to screen existing Kraft Heinz products for nutritional value and to set achievable and impactful targets for product reformulation that are category specific. Assuring transparency and disseminating the current and proposed nutrient standards is the purpose of the present report.

### 1.1. The Kraft Heinz Company global nutrition targets

The development of nutrition targets for innovation and renovation at KHC included setting target levels for the main nutrients of public health concern: saturated fat, total sugars, and sodium. Several key factors were considered, including the breadth of existing KHC products, their role in the diet, food ingredients, regulatory standards, technical considerations, taste, performance, and results from the benchmarking of foods.

Consideration was given to nutrient guidelines recommended by the WHO (19), Pan American Health Organization (20), the European Food Safety Authority (21), and the U.S. Food and Drug Administration (22). The nutrient daily values adopted by KHC were 20 g saturated fat, 50 g added sugar, 2,300 mg sodium and a mean daily energy value of 2,000 kcal, consistent with past practice (22). The applicability of each nutrient varies depending on the product category, for instance sodium is relevant to processed cheese, but not refreshment beverages (see Table 1).

**Table 1:** Global nutrition targets by The Kraft Heinz Company product category and subcategory.

^**1**^ **Product category and subcategory**	**Example/s**	^**2**^ **RACC** **(44)**	**kcal/** **100 g**	**SatFat** **g/100 g**	**TotSug** **g/100 g**	**Sodium mg/100 g**
**Sauces/Condiments**						
Tomato ketchup, regular and flavored	Curry Ketchup	1 tbsp	135	1.0	25.0	940
Spoonable dressings and mayonnaise	Tartar, Chipotle Aioli	15 g	670	10.0	13.5	830
Pourable salad dressings, oil based	Italian Dressing	30 g	230	3.5	16.5	1,165
Pourable dressings, cream based	Ranch Dressing	30 g	450	6.5	13.5	1,000
BBQ sauces	Curry Mango Sauce	1 tbsp	230	1.0	29	860
Mustards	Yellow Mustard	1 tsp	200	1.0	20	1,400
Dijon mustard	Dijon Mustard	1 tsp	200	1.0	20	2,400
Steak sauces, marinades, seasonings	Steak Sauce	1 tbsp	400	1.0	30	2,000
Soy sauce	Soy Sauce	1 tbsp	150	1.0	10	7,400
Oyster and fish sauce	Oyster Sauce	1 tbsp	120	1.0	20	4,840
**Meal Sauces**						
Meal and pasta sauces (Creamy)	Alfredo, Carbonara	125 g	150	4.0	3.5	665
Meal and pasta sauces (Oil Based)	Pesto Sauce	¼ cup	400	8.0	2.0	770
Meal and pasta sauces (Tomato)	Pasta and Pizza Sauce	125 g	80	1.0	7.0	370
Meal sauces (Gravy)	Finishing Sauces	¼ cup	70	3.5	2.0	560
Meal sauces (Asian)	Stir Fry, Satay Sauce	¼ cup	100	1.0	20	680
**Fruit, Vegetable, Legumes**						
Fruit packed in juice or syrup	Fruit Salad, Fruit Puree	140 g	75	1	12.5	150
Beans, legumes, vegetables with sauce	Beans in Sauce	130 g	100	1	8	350
Beans, legumes, vegetables with meat	Beans in Sauce & Meat	1 cup	115	1	6	350
Vegetables and legumes	Tomatoes, Corn, Beets	130 g	N/A	1	6^5^	150
**Meals**						
Single-food dishes (1 food group)	Pasta, Rice, French Fries	Varies	270	2.5	2.5^5^	530
Combination foods (≥1 food group)	Mac‘n Cheese, Pasta Salad	Varies	400	5^3^	10^5^	700^6^
Main dish (Center of plate)	Frozen Meal Centers, Pizza	Varies	200	3^4^	6	340
Meal type products	Ready Meals, Dinner Kits	Varies	210	3^4^	9	320
Soups	Canned/Chilled Soups	245 g	100	2	6.5	320
**Beverages**						
Refreshment beverages (<50% juice)	Capri Sun, Crystal Light	360 mL	50	N/A	9.5	N/A
Cordials	Liquid Concentrates	360 mL	40	N/A	8	N/A
Fruit and vegetable juice (≥50% juice)	Orange Juice, Tomato Juice	240 mL	90	N/A	12	N/A
Specialty beverages	Flavored Coffee	350 mL	50	1.5	4	N/A
Fruit nectars	Fruit Nectars	240 mL	70	N/A	14	N/A
Powdered drink mixes	Flavored Drink Mixes	Varies	375	N/A	75	N/A
**Cheese**						
Processed cheese	Processed Cheese Slices	30 g	460	19	12	1,730
Natural cheese	Brick, Sliced	30 g	665	23.5	N/A	1,200
Cream cheese	Cream Cheese	30 g	430	22	17	800
**Meat**						
Cooked bacon	Pork, Turkey Bacon	15 g	625	19	16	3,000
Dried meat	Salami, Pepperoni	40 g	460	11	9	1,710
Meat and meat substitutes	Chicken/Chik’n Strips	55 g	210	2.5	6	680
Processed meats and poultry	Luncheon Meat, Deli Meat	55 g	370	13	5	1,270
**Snacks**						
Bars, cookies and biscuits	Baked Biscuits, Cookies	40 g	430	9	34	570
Confections and chocolate	Marshmallows, Caramels	Varies	600	16	72	N/A
Spoonable sweet snacks	Pudding, Gelatin	½ cup	200	2	18	230
Powdered gelatin desserts	Powdered Gelatin	Varies	350	2	82	450
Nuts, nut butters and seeds	Peanut Butter	2 tbsp	665	12	27	660
Salted snacks	Corn Nuts, Crisps	30 g	500	3.5	3.5	1,165
Savory snack combinations	Pretzels and Cheese Dip	30 g	440	12	9	790
Sweet snack combinations	Cottage Cheese with Fruit	40 g	360	10	29	350
**Other**						
Dips and hummus	Non-sour Cream Dips	2 tbsp	335	7	10	570
Sweet bread toppings	Jam, Marmalade, Sprinkles	1 tbsp	350	1	N/A	125
Relish and chutney	Mix Vegetables Condiment	15 g	100	1	20	1,000
Pickles	Pickled Cucumbers	30 g	170	1	25	1,535
Desserts	Cheesecake, Pies	Varies	740	11	74	1,100
Fermented bean curd	Fermented Bean Curd	Varies	145	3	2	3,100

RACC, Reference Amounts Customarily Consumed; kcal, energy per 100g; SatFat, Saturated fats g/100g; TotSug, Total sugars g/100g; Sodium, Sodium mg/100g; N/A, not applicable.

1 Kraft Heinz products without nutrition targets include: salt, pepper, herbs and spices, yeast, tea, coffee, baking ingredients, vinegar and oil.

2 For measurement equivalents refer to the U.S. Department of Agriculture measurement conversion tables (44).

3 Add 2g saturated fat for items with cheese.

4 Add 1g saturated fat for items with cheese.

5 Exempt total sugar if no added sugar.

6 Add 100mg sodium for item requiring preparation with additional ingredients.

First, a category-specific approach was used based on the global KHC portfolio due to the wide variety of foods and beverages sold. Targets for nutrients of public health concern were developed for each product category. These included guidelines for optimal calories per 100 g (100 mL for beverages) and maximum amounts of sodium, total sugar, and saturated fat across all product lines, as described in Table 1. In the U.S., Reference Amounts Customarily Consumed (RACC) are established per eating occasion for labeling serving size (23). The RACC differs depending on food or beverage type. For example, condiments and sauces have a serving size between 5 and 30 g according to the U.S Food and Drug Administration RACC (23), whereas the RACC for a fruit or vegetable juice is 240 mL. As foods and beverages have various RACCs, the nutrient target varies depending on the specific type of food or beverage. For instance, the sodium targets per 100 g for a meal is more restrictive than salad dressing, as a meal is consumed in a larger quantity than the quantity used for a condiment. However, in other countries, serving size is not standardized and for this reason KHC uses the standardized amount of 100 g across all product lines.

Second, KHC nutrition targets had to be relevant to the dietary role of KHC products and to the eating occasion. The global nutrition targets reflect the dietary role of the product as outlined in national dietary guidelines (11, 24, 25). Dietary guidelines provide evidence-based recommendations on the types and amounts of foods that should be consumed to meet nutritional requirements. As such, consideration was given to the typical portion size consumed (26) as well as the way the product is consumed (meal, snack, beverage, and dessert), and whether the product contributes to discretionary calories.

Third, nutrient standards for a given product category are governed by the nutrient content within the category. The KHC global nutrition targets recognize that some ingredients can contribute vitamins and minerals along with nutrients of concern. For example, milk found in cheese and dairy products can be a source of both saturated fat and calcium. The sugar in 100% juice is considered naturally occurring in the fruit, while juice drinks may contain added sugars.

Fourth, some products are subject to regulatory standards, such as standards of identity adopted under Codex (27) and enforced under local laws, as in the case of cheeses, which are subject to standards dictating minimum fat and moisture levels (28). Another regulatory consideration are those required for front-of-pack labeling and nutrient content claims. For example, reduced sodium ketchup would need to contain a defined amount of sodium less than the reference amount, depending on local regulations.

Fifth there are technical challenges. For example, sodium has multiple functions in food, such as extending shelf life, stabilizing microbiological activity, enhancing flavor and palatability, and improving food structure and texture (29, 30). Therefore, its role in the food matrix may limit sodium reduction or replacement efforts. Reducing sodium in a product can be challenging when the mineral is present for reasons beyond taste, such as food safety or technical functions. Product innovations to reduce sodium content must guarantee food safety, be economically and technologically viable, and meet consumer sensory expectations (29). A change in fat content may also affect taste and functionality, including its intended use in recipes. For example fat emulsification is needed in pasteurized processed cheese to reinforce the structure of the product and maintain sensory characteristics (31). Lastly, the marketing and competitive set is relevant as the nutrient profiles of other products in the marketplace provide benchmarks for comparison, as described in Table 2.

**Table 2:** Comparison of The Kraft Heinz Company global nutrition targets with nutrient contents observed in the American (33) and French (34) markets for tomato ketchup, mayonnaise and French fries/fried potatoes.

	Number of products		Saturated fats (g/100 g)			Sodium (mg/100 g)			Total sugars (g/100 g)		
	U.S.	France	KHC target	U.S. (85^th^ %ile)	France (85^th^ %ile)	KHC target	U.S. (85^th^ %ile)	France (85^th^ %ile)	KHC target	U.S. (85^th^ %ile)	France (85^th^ %ile)
Tomato ketchup^1^	141	78	1	0	0.1	940	1,176	1,178	25	26.7	24
Mayonnaise^2^	202	124	10	14.3	9.0	830	833	640	13.5	6.7	1.9
French fries/fried potatoes^3^	259	123	2.5	1.8	1.7	530	435	138	2.5	1.2	0.6

KHC target, The Kraft Heinz Company global nutrition target; %ile, percentile.

1 Tomato ketchup, is classified within the Kraft Heinz product category ‘sauces and condiments’ and product subcategory ‘tomato ketchup’.

2 Mayonnaise, is classified within the Kraft Heinz product category ‘sauces and condiments’ and product subcategory ‘spoonable dressings and mayonnaise’.

3 French fries/fried potatoes, is classified within the Kraft Heinz product category ‘meals’ and subcategory ‘single-food side dishes’.

The KHC regional nutrition guidelines for leading markets, such as the United States, Europe, Australia, and New Zealand were used as a starting point to set the global nutrition targets. The KHC global nutrition targets (Table 1) are being used to guide new product development, set targets for reformulation and to monitor changes in the nutritional value of KHC product lines. Since 2019, the KHC global nutrition targets have been used to make environmental, social and governance (ESG) related commitments. In 2020, 74.7% of the KHC global portfolio met the KHC global nutrition targets (34). By 2025, KHC commits to improving product health and nutrition by achieving 85% compliance with Kraft Heinz global nutrition targets (35). The 85% target reflects an ambitious goal for setting the baseline of nutrition commitments, while balancing business priorities. This is a significant first step, however continuous work and reflections will be undertaken to extend these commitments in future years.

### 1.2. Benchmarking illustration for three Kraft Heinz products

Ketchup, mayonnaise and French fries/fried potatoes are popular foods worldwide and contribute markedly to KHC sales volume. These products were selected to illustrate how KHC global nutrition targets compare to the current market options available in the United States, France, and Australia.

Nutrient composition data for the United States market came from the U.S. Department of Agriculture (USDA) Branded Food Products Database (32). Items were selected based on a search of product names, duplicate entries were removed, and any products not fitting the category (e.g., potato chips) were also removed. The analytical samples were for ketchup, mayonnaise and potatoes, fried. Nutrient composition data for corresponding products in France came from the Agence Nationale Sécurité Sanitaire de l’Alimentation (ANSES) (33). Data for French fries were obtained as a weighted mean of 64 products for deep-frying, 50 for oven and 9 for micro-wave; for ketchup and mayonnaise, only regular products were included (i.e., no sugar-reduced ketchup or fat-reduced mayonnaise). Of note, database values are not weighted relatively to market shares and may inadequately reflect the reality of nutrient contents. Table 2 compares KHC nutrition targets for ketchup, mayonnaise, and French fries with the 85^th^ percentile cut-points of the distribution for currently available products. For a normal distribution, the 85^th^ percentile is one standard deviation above the mean.

KHC target for sodium in ketchup was well below the 85^th^ percentile cut-point in both United States and French databases. For mayonnaise, KHC sodium target was below the cut-point in the United States but above the cut-point in French data. For French fries, the KHC sodium target was above the cut-points both in the United States and in French databases. KHC target for added sugar in ketchup was at ~85^th^ percentile cut-point for both data sets; by contrast KHC targets for added sugar in mayonnaise and French fries were substantially higher. The KHC target for saturated fat in mayonnaise was below the United States but above the French cut-point.

These data illustrate the difficulty of reformulating products across different markets and product lines. First, based on our analyses of nutrient composition databases, these products in France contained lower amounts of saturated fats, sodium, and total sugars. Whether this was related to diet quality is not clear, since dietary intake data was not available. Second, product categories are not always totally comparable across different markets. For example, KHC nutrition targets are lower than observed values in 15% of products for sodium in ketchup and, in the United States only, for sodium and saturated fats in mayonnaise and sugar in ketchup. The KHC sugar target for mayonnaise is above the market reality, as the KHC subcategory for spoonable dressings and mayonnaise includes sauces other than mayonnaise (e.g., tartar sauce and chipotle aioli) that may contain sugar. Reassessing the targets for spoonable dressings and mayonnaise could be considered for the future.

Currently there is no publicly available Australian branded food database for use by consumers, researchers, or businesses. As such, the George Institute for Global Health in Australia FoodSwitch smartphone app (36) was used to identify a list of healthier choices, as determined by the Health Star Rating (37). Interestingly, when assessing Australian products *via* the Foodswitch app, it was observed that 20% of ketchups (21 out of a total of 103, assessed on the 14 June 2022) had greater than or equal to 3.5 health stars and therefore classified by ATNI as a “healthy” product (18). On assessment, these better for you ketchups (*n* = 21) had <700 mg sodium and <24 g sugars per 100 g.

## 2. Discussion: New directions in product innovation and reformulation

Product reformulation and innovation provides an opportunity for the food industry to improve the nutritional value of packaged foods. Research suggests that dietary behavior change observed in some consumers following product reformulation does not offset the overall benefits of product reformulation on dietary intakes at the population level (1). Indeed, reformulation programs implemented across and within all food categories can improve diet quality (1).

The WHO’s recommendation is to achieve a 30% relative reduction in mean population intake of sodium by 2025 (38). The WHO also advises limiting total sugar to 90 g per day and free sugar (i.e., added sugar, as well as sugars in honey, syrups and fruit juices) to 50 g per day. Sugar is added to products for functional properties including enhancing flavor and sweetness, improving mouthfeel and texture, color formation, fermentation and preservation (39). Sugar also plays an important role in enjoyment and pleasure of the product, given its sweet perception.

Sensory and consumer research are essential in product innovation. This is to ensure that reformulated foods will meet consumers’ expectations and be consumed, and therefore have a dietary impact and public health benefit. Sensory evaluation is a science that measures the reactions to products as perceived by sight, smell, taste, touch and hearing (40). Different types of sensory evaluation methods are required to determine consumers’ preferences for new products (41). Food choice is complex, so it is critical in the product development cycle to understand not only sensory factors, but other factors including consumer attitudes, emotions, behaviors and the context in which the new food will be consumed (41).

Another aspect of product (re)formulation is an increase in positive nutrients and/or food components (e.g., fiber, polyunsaturated fats, and whole food ingredients such as whole grains, nuts, legumes, seeds, vegetables and fruits) to improve overall nutrient density.

Product formulation can be an efficient means of optimizing both nutrient content and nutrient bioavailability within a food. For instance, adding oil to tomatoes favors the absorption of lycopene, a potent antioxidant carotenoid present in high amounts in tomatoes (42). Additionally, the thermal and mechanical techniques used in food processing allows for lycopene to become more bioavailable during digestion and absorption (43).

KHC’s 2025 ESG nutrition commitments include: achieve 85% compliance with KHC’s global nutrition targets, reduce total sugar in products by more than 60 million pounds across the global portfolio and reduce sodium by 5% in Kraft BBQ sauce and salad dressings, in North America (34).

As one example of sugar reduction, in August 2022, KHC’s CapriSun in the United States and Canada, launched reformulated juice drink pouches with an average of 40% less sugar than the original product. Each 6 fl. oz. serving (177 mL) now has an average of 8 g of total sugars and 5 g of added sugars. The sugar reduction in CapriSun accounts for more than half of the proposed sugar commitment.

Key nutrition priorities for KHC over the coming years are to continue to gradually reduce nutrients of public health concern (especially sodium and sugar) and increase positive nutrients and food groups, while considering consumer demand related to taste and texture. Furthermore, the benchmarking of products additional to those already presented, that make up the major volume of sales of KHC could suggest improvements of global nutrition targets. For maximum public health benefit, the planned gradual improvements in nutrient density need to occur without provoking major changes in consumer food choice and purchasing behavior.

Where feasible, KHC can also explore including nutrients (dietary fiber, protein, vitamins and minerals) and food groups to encourage in the global nutrition targets. Current examples of products with food groups to encourage include 100% whole wheat macaroni (*Mac and Cheese*), vegetable and legume based soups with 15 g of plant protein per serve (*Heinz Plant Proteinz*), *Classico* tomato sauces with ½ cup of vegetables per serving from tomatoes, meal-type products (*SmartOnes* entrees), containing ½ cup vegetables per serving, and whole-grain offerings from *Lunchables* products in the form of crackers.

KHC global nutrition targets represent a set of feasible standards for product innovation and renovation. Efforts at product (re)formulation will have a global reach since these nutrition targets are being applied to the KHC’s global food and beverage portfolio. The present examples from branded food composition datasets from the United States and France show that the targets are being met by products among the major volume of sales of KHC; ketchup, mayonnaise and French fried potatoes. Improving nutrient composition of frequently eaten foods has the potential to improve overall dietary intake. Further enhancements of the KHC product portfolio will involve the continued reduction in sodium and sugar content and the addition of wholesome food components to packaged foods. There is also potential to transform the existing global nutrition targets into a KHC nutrient density score.

## Data availability statement

The original contributions presented in the study are included in the article, further inquiries can be directed to the corresponding author.

## Author contributions

All authors contributed to the manuscript design, analysis of data, writing and approval of the final manuscript.

## Funding

This study was fully funded by The Kraft Heinz Company.

## Conflict of interest

LY, BE, and AB were full time employees of The Kraft Heinz Company during the manuscript preparation and submission. VB is a member of The Kraft Heinz Global Nutrition Advisory Group and the owner of VAB-nutrition, a consultancy company providing nutrition-related scientific support to food industries. SC is a member of The Kraft Heinz Global Nutrition Advisory Group and provides consulting services to industry with interests in food labeling and sustainable diets. AD is the original developer of the Nutrient Rich Food (NRF) index, a nutrient profiling model. That work was supported at the time by the Nutrient Rich Coalition whose members were The Beef Checkoff Program through the National Cattlemen’s Beef Association, California Avocado Commission, California Kiwifruit, California Strawberry Commission, Egg Nutrition Center, Florida Department of Citrus, Grain Foods Foundation, National Dairy Council, National Pork Board, United States Potato Board, Wheat Foods Council, and Wild Blueberry Association of North America. AD is a member of Nestlé Scientific Advisory Board and invited member of the Quality Carbohydrate Coalition supported by APRE and Potatoes USA. AD is a member of The Kraft Heinz Global External Nutrition Advisory Group. AD has received grants, contracts, and honoraria from entities both public and private with an interest in nutrient density metrics and nutrient profiling of foods.

## Publisher’s note

All claims expressed in this article are solely those of the authors and do not necessarily represent those of their affiliated organizations, or those of the publisher, the editors and the reviewers. Any product that may be evaluated in this article, or claim that may be made by its manufacturer, is not guaranteed or endorsed by the publisher.

## References

[1] Gressier M Swinburn B Frost G Segal AB Sassi F. What is the impact of food reformulation on individuals' behaviour, nutrient intakes and health status? A systematic review of empirical evidence. Obes Rev. (2021) 22:e13139. doi: 10.1111/obr.13139, PMID: 33022095

[2] Wahlqvist ML Wattanapenpaiboon N. Part 3: food, nutrients and other bioactive food components In: Gallegos D Wahlqvist ML, editors. Food and nutrition: sustainable food and health systems. 4th ed. London: Taylor & Francis Group (2020).

[3] Dietary Guidelines Advisory Committee. Scientific Report of the 2020 Dietary Guidelines Advisory Committee: Advisory Report to the Secretary of Agriculture and the Secretary of Health and Human Services. Washington, DC: U.S. Department of Agriculture, Agricultural Research Service (2020). Available from: https://www.dietaryguidelines.gov/sites/default/files/2020-07/ScientificReport_of_the_2020DietaryGuidelinesAdvisoryCommittee_first-print.pdf) (Accessed September 5, 2022).

[4] World Health Organisation. *Reformulation of Food and Beverage Products for Healthier Diets: Policy Brief* (2022). Available from: https://www.who.int/publications/i/item/9789240039919

[5] GDB 2017 Diet Collaborators. Health effects of dietary risks in 195 countries, 1990-2017: a systematic analysis for the global burden of disease study 2017. Lancet. (2019) 393:1958–72. Epub 2019/04/08. doi: 10.1016/s0140-6736(19)30041-8, PMID: 30954305 PMC6899507

[6] Public Health England. *Salt Reduction Targets for 2024* (2020). Available from: https://www.gov.uk/government/publications/salt-reduction-targets-for-2024 (Accessed September 5, 2022).

[7] U.S. Food and Drug Administration. *Guidance for Industry: Voluntary Sodium Reduction Goals* (2021). Available from: https://www.fda.gov/regulatory-information/search-fda-guidance-documents/guidance-industry-voluntary-sodium-reduction-goals (Accessed September 22, 2022).

[8] Ministry of Agriculture and Food Sovereignty. *National Food Program 2019–2023: Territories in Action* (2020). Available from: https://agriculture.gouv.fr/programme-national-pour-lalimentation-2019-2023-territoires-en-action (Accessed September 5, 2022).

[9] Department of Health and Aged Care. *Partnership Reformulation Program–Summary of Food Categories and Reformulation Targets: Commonwealth of Australia* (2021). Available from: https://www.health.gov.au/resources/publications/partnership-reformulation-program-summary-of-food-categories-and-reformulation-targets

[10] Downs SM Thow AM Leeder SR. The effectiveness of policies for reducing dietary trans fat: a systematic review of the evidence. Bull World Health Organ. (2013) 91:262–269H. doi: 10.2471/BLT.12.111468, PMID: 23599549 PMC3629452

[11] U.S. Department of Agriculture and U.S. Department of Health and Human Services. *Dietary Guidelines for Americans, 2020–2025*. 9th ed. (2020). Available from: https://www.dietaryguidelines.gov/ (Accessed September 5, 2022).

[12] Lehmann U Charles VR Vlassopoulos A Masset G Spieldenner J. Nutrient profiling for product reformulation: public health impact and benefits for the consumer. Proc Nutr Soc. (2017) 76:255–64. Epub 2017/04/20. doi: 10.1017/s0029665117000301, PMID: 28420455

[13] Vlassopoulos A Masset G Charles VR Hoover C Chesneau-Guillemont C Leroy F. A nutrient profiling system for the (re)formulation of a global food and beverage portfolio. Eur J Nutr. (2017) 56:1105–22. Epub 2016/02/18. doi: 10.1007/s00394-016-1161-9, PMID: 26879847 PMC5346408

[14] Roodenburg AJC. Nutrient profiling for front of pack labelling: how to align logical consumer choice with improvement of products? Proc Nutr Soc. (2017) 76:247–54. Epub 2017/09/01. doi: 10.1017/s0029665117000337, PMID: 28857018

[15] Roodenburg AJ Schlatmann A Dötsch-Klerk M Daamen R Dong J Guarro M. Potential effects of nutrient profiles on nutrient intakes in the Netherlands, Greece, Spain, USA, Israel, China and South-Africa. PLoS One. (2011) 6:e14721. Epub 2011/03/05. doi: 10.1371/journal.pone.0014721, PMID: 21373186 PMC3044133

[16] Greenberg D Drewnowski A Black R Weststrate JA O'Shea M. A progressive nutrient profiling system to guide improvements in nutrient density of foods and beverages. Front Nutr. (2021) 8:774409. Epub 2022/01/11. doi: 10.3389/fnut.2021.774409, PMID: 35004807 PMC8733001

[17] Furuta C Jinzu H Cao L Drewnowski A Okabe Y. Nutrient profiling of japanese dishes: the development of a novel ajinomoto group nutrient profiling system. Front Nutr. (2022) 9:912148. Epub 2022/08/16. doi: 10.3389/fnut.2022.912148, PMID: 35967784 PMC9372512

[18] Access to Nutrition Initiative. *Global Access to Nutrition Index 2021-Methodology* (2020). Available from: https://accesstonutrition.org/app/uploads/2020/06/Global-Index-2021-Methodology-FINAL.pdf (Accessed July 30, 2022).

[19] World Health Organization. *Guideline: Sugars Intake for Adults and Children* (2015). Available from: https://www.who.int/publications/i/item/9789241549028 (Accessed October 10, 2022). 25905159

[20] Pan American Health Organization. *Updated Paho Regional Sodium Reduction Targets: A Tool to Tackle the Burden of Diet-Related Noncommunicable Diseases* (2021). Available from: https://iris.paho.org/bitstream/handle/10665.2/55001/PAHONMHRF210013_eng.pdf?sequence=4&isAllowed=y (Accessed October 1, 2022).

[21] EFSA Panel on Nutrition, Novel Foods and Food Allergens (NDA) Turck D Castenmiller J de Henauw S Hirsch-Ernst K-I Kearney J. Dietary reference values for sodium. EFSA J. (2019) 17:e05778. doi: 10.2903/j.efsa.2019.5778, 32626425 PMC7009309

[22] U.S. Food and Drug Administration. *Daily Value and Percent Daily Value: Changes on the New Nutrition and Supplement Facts Labels* (2020). Available from: https://www.fda.gov/media/135301/download (Accessed October 10, 2022).

[23] U.S. Food and Drug Administration. *Reference Amounts Customarily Consumed: List of Products for Each Product Category: Guidance for Industry* (2018). Available from: https://www.fda.gov/media/102587/download (Accessed September 22, 2022).

[24] National Health and Medical Research Council. *Australian Dietary Guidelines* (2013). Available from: https://www.nhmrc.gov.au/adg (Accessed July 15, 2022).

[25] Sante Publique France. *Recommandations Sur L’alimentation, L’activité Physique & La Sédentarité Pour Les Adultes* (2019). Available from: https://www.santepubliquefrance.fr/presse/2019/sante-publique-france-presente-les-nouvelles-recommandations-sur-l-alimentation-l-activite-physique-et-la-sedentarite (Accessed October 17, 2022).

[26] Livingstone MBE Pourshahidi LK. Portion size and obesity. Adv Nutr. (2014) 5:829–34. doi: 10.3945/an.114.007104, PMID: 25398749 PMC4224223

[27] Food and Agriculture Organization/World Health Organisation. *Codex Alimentarius International Food Standards* (2022). Available from: https://www.fao.org/fao-who-codexalimentarius/codex-texts/list-standards/en/ (Accessed October 16, 2022).

[28] Food and Agriculture Organization. *Gateway to Dairy Production and Products* (2019). Available from: https://www.fao.org/dairy-production-products/products/codex-alimentarius/en/ (Accessed October 16, 2022).

[29] Barcenilla C Álvarez-Ordóñez A López M Alvseike O Prieto M. Microbiological safety and shelf-life of low-salt meat products-a review. Foods. (2022) 11:1–24. doi: 10.3390/foods11152331 PMC9367943 35954097

[30] Buttriss JL. Food reformulation: the challenges to the food industry. Proc Nutr Soc. (2013) 72:61–9. Epub 2012/12/12. doi: 10.1017/s0029665112002868, PMID: 23228239

[31] Shirashoji N Jaeggi JJ Lucey JA. Effect of trisodium citrate concentration and cooking time on the physicochemical properties of pasteurized process cheese. J Dairy Sci. (2006) 89:15–28. doi: 10.3168/jds.S0022-0302(06)72065-3, PMID: 16357264

[32] U.S. Department of Agriculture Ag Data Commons. *Usda Branded Food Products Database*. (2022). Available from: https://data.nal.usda.gov/dataset/usda-branded-food-products-database (Accessed September 18, 2022).

[33] ANSES. *Simulation De Seuils De Reformulation Par Famille D’aliments Transformés Et Impact Sur Les Apports En Sucres, Acides Gras Saturés, Sel Et Fibres De La Population Française* (2021). Available from: https://www.anses.fr/en/system/files/UOA2019SA0122Anx.pdf (Accessed September 5, 2022).

[34] Kraft Heinz Company. *Together at the Table: Kraft Heinz 2022 Esg Report* (2022). Available from: https://www.kraftheinzcompany.com/esg/pdf/KraftHeinz-2022-ESG-Report.pdf (Accessed October 18, 2022).

[35] Kraft Heinz Company. *Product Health and Nutrition Global Nutrition Guidelines* (2022). Available from: https://www.kraftheinzcompany.com/esg/nutrition-guidelines.html (Accessed October 10, 2022).

[36] The George Institute for Global Health Australia. *Foodswitch* (2022). Available from: https://www.georgeinstitute.org.au/projects/foodswitch (Accessed October 17, 2022).

[37] Commonwealth of Australia. *Health Star Rating System* (2022). Available from: http://www.healthstarrating.gov.au/internet/healthstarrating/publishing.nsf/Content/Home (Accessed October 17, 2022).

[38] World Health Organization. Global Action Plan for the Prevention and Control of Noncommunicable Diseases 2013–2020. Geneva, Switzerland: World Health Organization (2013) Available from: https://www.who.int/publications/i/item/9789241506236.

[39] Goldfein KR Slavin JL. Why sugar is added to food: food science 101. Compr Rev Food Sci Food Saf. (2015) 14:644–56. doi: 10.1111/1541-4337.12151

[40] Stone H. Example food: what are its sensory properties and why is that important? NPJ Sci Food. (2018) 2:11. doi: 10.1038/s41538-018-0019-3, PMID: 31304261 PMC6550172

[41] Ruiz-Capillas C Herrero AM Pintado T Delgado-Pando G. Sensory analysis and consumer research in new meat products development. Foods. (2021) 10:1–15. doi: 10.3390/foods10020429 PMC7919803 33669213

[42] Gärtner C Stahl W Sies H. Lycopene is more bioavailable from tomato paste than from fresh tomatoes. Am J Clin Nutr. (1997) 66:116–22. Epub 1997/07/01. doi: 10.1093/ajcn/66.1.116, PMID: 9209178

[43] Arballo J Amengual J Erdman JW. Lycopene: a critical review of digestion, absorption, metabolism, and excretion. Antioxidants. (2021) 10:342. doi: 10.3390/antiox10030342, PMID: 33668703 PMC7996133

[44] U.S. Department of Agriculture. *Measurement Conversion Tables* (2020). Available from: https://www.ars.usda.gov/northeast-area/beltsville-md-bhnrc/beltsville-human-nutrition-research-center/methods-and-application-of-food-composition-laboratory/mafcl-site-pages/measurement-conversion-tables/ (Accessed October 20, 2022).

